# Are left-behind families of migrant workers at increased risk of attempted suicide? – a cohort study of 178,000+ individuals in Sri Lanka

**DOI:** 10.1186/s12888-018-2000-8

**Published:** 2019-01-15

**Authors:** Duleeka Knipe, Helen Lambert, Melissa Pearson, Michael Eddleston, Shaluka Jayamanne, Kolitha Wickramage, Keith Hawton, Flemming Konradsen, Chris Metcalfe, David Gunnell

**Affiliations:** 1 South Asian Clinical Toxicology Research Collaboration (SACTRC), Faculty of Medicine, University of Peradeniya, Peradeniya, Sri Lanka; 2 Population Health Sciences, Bristol Medical School, University of Bristol, Canynge Hall, 39 Whatley Road, Bristol, BS8 2PS UK; 3 Pharmacology, Toxicology & Therapeutics, University/BHF Centre for Cardiovascular Science, University of Edinburgh, Edinburgh, UK; 4 Centre for Pesticide Suicide Prevention, University of Edinburgh, Edinburgh, UK; 5 Faculty of Medicine, University of Kelanyia, Kelanyia, Sri Lanka; 6 Migration Health Division, International Organization for Migration, UN Migration Agency, Migration Health Centre, Manila, Philippines; 7 Centre for Suicide Research, Department of Psychiatry, University of Oxford, Oxford, UK; 8 Department of Public Health, Faculty of Health and Medical Sciences, University of Copenhagen, Copenhagen, Denmark; 9 National Institute for Health Research Bristol Biomedical Research Centre, University Hospitals Bristol NHS Foundation Trust and University of Bristol, Bristol, England

**Keywords:** Migration, Left-behind children, Sri Lanka, Asia, Suicide

## Abstract

**Background:**

There are an estimated 258 million international migrants worldwide. In Asia low-skilled workers often emigrate on a temporary basis (2–3 years) without their families. There is significant concern over the mental health and wellbeing of left-behind families in this region. No previous study has examined whether the risk of suicidal behaviour is elevated in left-behind family members.

**Methods:**

Cohort study using baseline data from a large randomised controlled trial in Sri Lanka (*n* = 178,730 participants; 8% households had a current temporary foreign migrant) and prospective hospital presentations of suicide attempts. Using multilevel Poisson regression models, we compared the risk of attempted suicide in households with left-behind and non-left-behind family members. We also investigated whether the sex of the migrant or the age/sex of the household member left behind altered any associations.

**Results:**

The risk of an attempted suicide was elevated in female migrant households (IRR 1.60 95% CI 1.38, 1.85), but not male migrant households (IRR 1.01 95% CI 0.76,1.36)) with strong evidence that risk differed for female vs. male migrant households (*p*-value = 0.005). We found no evidence that the age or sex of the left-behind household member altered the association observed.

**Conclusions:**

This analysis suggests that members of households with a temporary female foreign migrant are at an increased risk of attempted suicide, but these findings must be interpreted with caution. The increased risk of suicidal behaviour in these households may be due to factors that were present before the migration and persist post-migration (e.g. household violence, poverty).

**Electronic supplementary material:**

The online version of this article (10.1186/s12888-018-2000-8) contains supplementary material, which is available to authorized users.

## Background

Globalisation has brought about profound societal changes in low and middle-income countries (LMIC). Dramatic increases have been observed in international migration from LMIC, with an estimated 164 million international migrants worldwide from this part of the world out of 258 million migrants globally [1]. Forty-percent of these migrants are from Asia. Migration is an important driver for development in LMICs through the provision of foreign remittances. However, this migration occurs within the context of stringent migration policies (e.g. restrictions on the migrant’s family accompanying them), which has led to a growing number of transnational families, with millions of left-behind children in countries of origin when parent(s) migrate for temporary (2–3 years) work abroad [2, 3]. Many of the women who migrate in this way tend to be young mothers.

Some evidence suggests that temporary foreign migration has negative health consequences on family members left behind, with offspring having increased behavioural/emotional problems; impaired cognitive development; underage marriages; and experiences of child abuse [4–11]. Yet, other evidence suggests a positive or no impact on offspring [8, 12–15]. There are limitations to this evidence, however, such as the temporal relationship between migration and the social and health circumstances of families not being established by the cross-sectional and retrospective designs used in many of the research studies, the potential for confounding of associations in observational studies, and the modest sample sizes in some studies which may be insufficient to reliably identify associations (low statistical power).

A recent systematic review of the health consequences of parental migration on left-behind children and adolescents concluded that there is a paucity of evidence from LMIC on the impact of emigration on left-behind family members [16]. The review called for more longitudinal studies from LMIC investigating the impact of emigration.

Using a large prospective cohort study in rural Sri Lanka we aimed to answer the following questions: i) Is there an increased risk of suicidal behaviour in families left-behind by temporary foreign migrants?; ii) Is there evidence that any associations differ depending on the sex of the migrant?; and iii) Does the impact of the migration differ by age or sex of the left-behind family members?

## Methods

### Setting

Sri Lanka is a lower middle-income country situated off the south-east coast of India with a population of 21 million (Census 2011). Nearly 80 % of the population live in rural areas and nearly a third are employed in agriculture. In the late 1970s, as a consequence of the open economy reforms (facilitating freer migration flows) and a Middle East oil boom, a large number of non-skilled temporary work opportunities became available for Sri Lankans [17]. Despite the temporary nature of the jobs, the financial incentives and lack of local alternatives for individuals from a lower socioeconomic position (SEP) meant a large number of Sri Lankans sought work overseas, especially women [4, 17]. This trend continues to this day, with nearly 300,000 (male: 172,788; female: 90,655) Sri Lankans departing for foreign employment in 2015, although the number of male migrants now exceeds females [18]. The current study was conducted in the Anuradhapura district, a rural part of the country where nearly 12,000 (1.3% of the population) (male: 5382; female: 6312) individuals emigrated for work in 2015 [18].

### Participants

The cohort is based on participants of a large cluster randomised controlled trial (RCT) in rural Sri Lanka [19]. The RCT evaluated the effectiveness of lockable storage boxes in reducing self-poisoning with pesticides in a rural area of the Anuradhapura District between December 2010 and May 2016 and found no effect on risk of suicidal behaviour. The details of data collection for the trial have been described previously [20, 21] and are provided here in brief. The trial included a baseline door-to-door survey with all eligible households in the study area between December 2010 and February 2013. An adult member of the household (≥18 years) was interviewed (face-to-face) and detailed demographic information collected on each household member after verbal consent was obtained. The survey tool is described elsewhere [22]. Individuals who would normally be resident in the household, but were temporarily absent were included, and in this way data on migrants were obtained.

### Variables

#### Exposure

The baseline survey included a ‘foreign employed’ occupation category for each household member but, due to time/resource constraints, we were unable to collect data on the type (e.g. skilled vs. unskilled) of migration. Official interest in Sri Lanka is focussed on temporary migration of individuals for non-skilled work (e.g. domestic labour), as this is the main type of migration from Sri Lanka. For this analysis we therefore identified households with a non-graduate (i.e. no university qualification) foreign employed individual (referred to as ‘migrant households’ henceforth) at the time of the baseline survey. Only 0.8% of households had a graduate foreign employed household member, compared to 8% of households with a non-graduate foreign employed household member. Graduate migrants don’t tend to experience the same stringent migration restrictions as their non-graduate counterparts, and the drivers for this type of migration are likely to be different to unskilled emigration. We therefore excluded graduate migrants from the analysis. We further categorised migrant households according to the sex of the migrant/s (male, female, or both sexes).

#### Outcome

Data were collected on incident episodes of suicide and attempted suicide presenting to hospital (see [19]). Our primary outcome was attempted suicide. For the larger trial, we identified 2882 suicide attempts and 188 suicide deaths; 2371 (82%) of attempts and 160 (85%) of suicide deaths were linked back to an individual in the baseline dataset. Given the staggered nature of baseline data collection, participants were followed up for different periods of time (3–5 years; median 3.6 years). To check that we captured all the suicide attempts and deaths that occurred in the study area the field team revisited 26% of households to ask about any suicide attempts that had occurred since the baseline survey. These households were randomly selected.

#### Potential confounders/modifiers/mediators

The other variables included in this analysis were age, sex, and intervention arm. These were considered as confounders in the first set of models (see below), and age and sex were considered as modifiers in the second set of models. Age was categorised into 4 age groups 10–25; 26–40; 41–55; and 56+ years of age. These age groups were based on the age-specific incidence of suicide attempts within the dataset. In the main trial findings there were no differences between the risk of attempted suicide between the two arms of the trial [19], but we have taken the conservative approach of adjusting for intervention arm in our analysis. We also explored the effect of controlling for other potential confounders/mediators but these were not included as part of the main analysis as the relationship of these factors (‘problem’ alcohol use, household SEP and household size) with the exposure and outcome of interest is unclear (i.e. whether they were causes or consequences of the migration).

### Statistical analysis

**Fig. 1:**
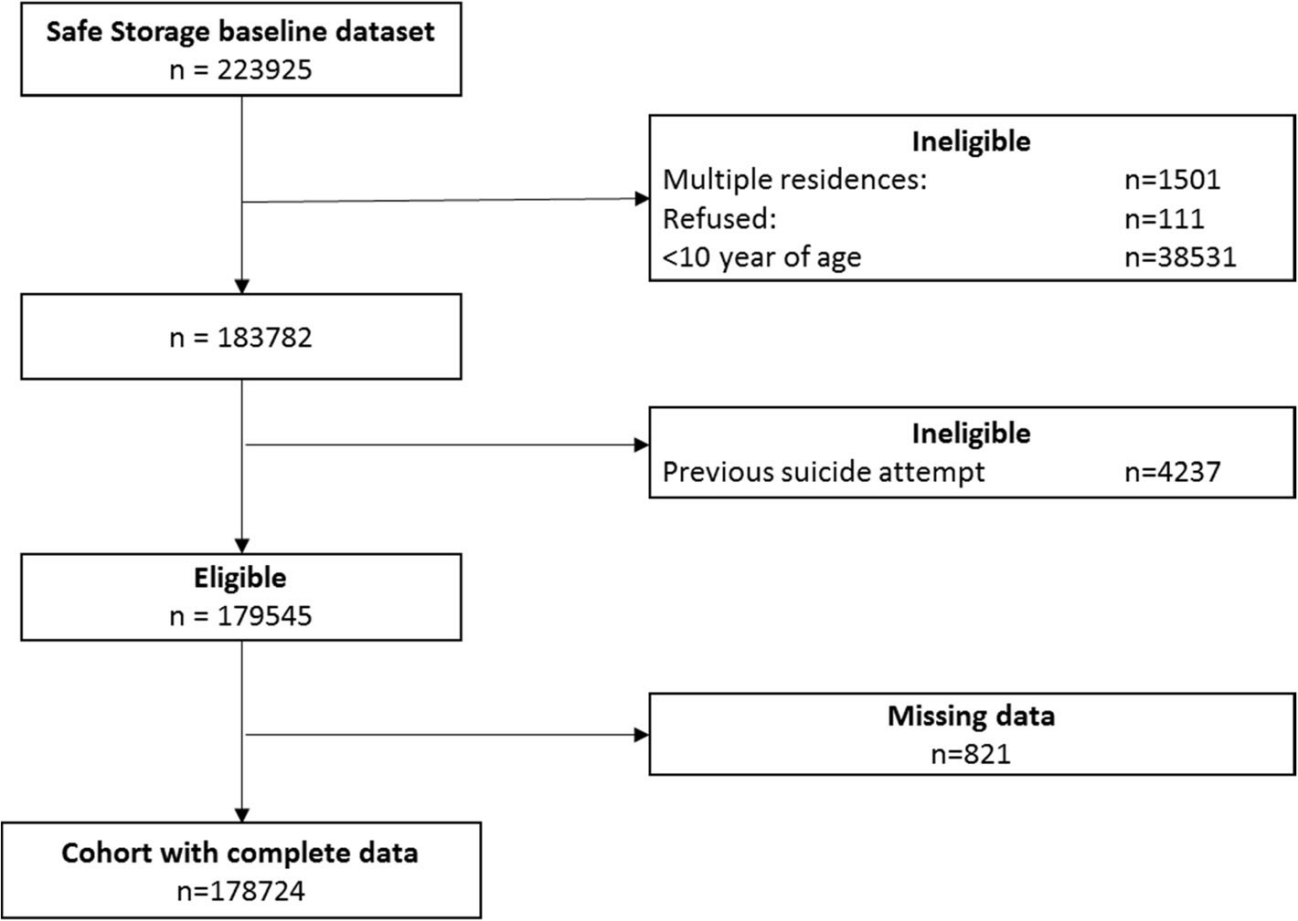
Flow chart of numbers of individuals included in the cohort analysis

This is a secondary analysis of a dataset. The data are clustered in nature, with individuals living within households, within communities. For the purpose of this analysis we have used the same cluster boundaries which were used in the trial to define communities [23]. We fitted mixed effects Poisson regression models (multi-level models) accounting for clustering at household and community level. Total available follow-up time was used as the offset, having taken account of the variable frequency at which individuals were present in that household. We considered household members of age ten years and older as at risk of suicidal behaviour, i.e. we assumed children younger than ten years would not intentionally harm themselves. We also excluded those who had a respondent-reported attempted suicide prior to the baseline survey (Fig. 1) to ensure that the cohort were outcome free at the start of the study.

The main analysis included two sets of models investigating the association between living in a migrant household and suicide attempt risk: i) age- (categorical variable), sex- and intervention arm-adjusted models for household migration status (non-migrant, female migrant, male migrant and both male and female migrant households); and ii) age category- (defined above) and sex-stratified models of those left behind with a test for interaction. We did this by fitting models with and without an interaction parameter for age and sex separately (age was treated as a continuous variable for just these models), and then tested which of the two models was a better fit for the data using the likelihood ratio test. We present the *p*-value for interaction and the results stratified by sex and age group. We explored the effect of controlling for three additional variables (education level of the head of household, ‘problem’ alcohol use and household size) as a secondary analysis. We also explored the association between being left behind and suicide risk (secondary outcome).We conducted a complete case analysis, excluding participants with missing data for the variables included in this analysis (Additional file 1). As a sensitivity analysis we included graduate foreign migrant households in our migrant household category. We present the unadjusted associations in a supplementary file (Additional file 1).

### Ethics, consent and permissions

Ethical approval was received from the research ethics committees of the University of Peradeniya and Rajarata University of Sri Lanka. This was a study was a secondary analysis of data collected as part of a larger randomised controlled trial which was registered with ClinicalTrials.gov, number NCT1146496.

## Results

### Population characteristics

Of the 223,925 individuals included in the baseline survey, 178,724 participants were included in this analysis (Fig. 1). Individuals with missing data (0.5% of the sample – Fig. 1) were more likely to be older, have lower levels of education, and spend less time at home. Ten percent of individuals (*n* = 18,140), from 8% of study households, lived in migrant households (Table 1). Left-behind individuals in female migrant households were more likely to be female, younger, and have a head of household with a lower level of education than non-migrant households. Individuals in male migrant households were slightly more likely to be male, and young adults (26–40 year old). Most of the migrant households had a female migrant (*n* = 3014, 67%), with a smaller number having only a male migrant (*n* = 1268 28%), or both male and female migrants (*n* = 201, 5%). The mean age of migrants was 35 years (SD 9.2), with female migrants being slightly older on average than male migrants (females: 36 years (SD 9.4); males: 33 years (SD 8.5).

**Table 1:** Characteristics of study cohort by household migrant status

	Migration status of household n (%)			
	Non-migrant	Female-migrant	Male-migrant	Female & Male-migrant
N=	160,584 (89.9)	11,972 (6.7)	5118 (2.9)	1050 (0.6)
Household measures				
Problem’ alcohol use	42,207 (26.3)	3320 (27.7)	1057 (20.7)	283 (27.0)
Education of head of household				
University	1886 (1.2)	23 (0.2)	32 (0.6)	0 (0)
A-level	17,870 (11.1)	429 (3.6)	504 (9.8)	51 (4.9)
O-Level	96,086 (59.8)	6347 (53.0)	3249 (63.5)	544 (51.8)
Primary	39,329 (24.5)	4444 (37.1)	1177 (23.0)	408 (38.9)
Not attended	5413 (3.4)	729 (6.1)	156 (3.0)	47 (4.5)
Household size median (IQI)^a^	4 (3–5)	4 (3–5)	4 (3–5)	6 (5–7)
Individual measures				
Sex				
Female	81,305 (50.6)	6704 (56.0)	2366 (46.2)	542 (51.6)
Male	79,279 (49.4)	5268 (44.0)	2752 (53.8)	508 (48.4)
Age group (years)				
10–25	49,211 (30.6)	3884 (32.4)	1369 (26.7)	308 (29.3)
26–40	50,117 (31.2)	3633 (30.3)	1873 (36.6)	381 (36.3)
41–55	35,684 (22.2)	2728 (22.8)	1044 (20.4)	193 (18.4)
> 55	25,572 (15.9)	1727 (14.4)	832 (16.3)	168 (16.0)
Frequency at home				
< 30 days	3874 (2.4)	2191 (18.3)	911 (17.8)	312 (29.7)
1–6 months	25,929 (16.1)	2326 (19.4)	955 (18.7)	217 (20.7)
7–11 months	21,116 (13.1)	1663 (13.9)	581 (11.4)	120 (11.4)
Always	109,665 (68.3)	5792 (48.4)	2671 (52.2)	401 (38.2)
Attempted suicide n (per 100,000)^b^	1782 (324.9)	220 (691.1)	51 (363.2)	7 (304.6)
Suicide deaths n (per 100,000)^b^	103 (18.7)	13 (4.0)	3 (2.1)	1 (4.3)

^a^*IQI* Interquartile interval

^b^Annual attempted suicide and suicide deaths rate (prospective surveillance data)

### Results from the primary analysis

There were 2060 suicide attempts in the 3–5-year follow-up period. The annual suicide attempt rate was higher in migrant (577 per 100,000, 95% CI 513, 649 per 100,000) than non-migrant households (325 per 100,000, 95% CI 310, 340 per 100,000). The suicide attempt rate was highest in female migrant households (691 per 100,000, 95% CI 606, 789 per 100,000) (Table 1).

Living in a female migrant household was associated with a 60% (95% CI 38, 85%) increased risk of attempted suicide, whereas there was no statistical evidence of an elevated risk in male migrant households (Table 2). There was strong statistical evidence that the risk was different in female vs. male migrant households (*p* = 0.005). The effect estimates were slightly larger in male and older left-behind family members, but there was no statistical evidence to suggest that the associations observed were differed by the sex or age of the left-behind family member.

**Table 2:** Age and sex adjusted risk of attempted suicide by household (hh) migrant status

	Age & Sex adjusted^a^ - IRR (95% CI)	Sex of household member^b^ - IRR (95% CI)		Age of household member stratified^c^ - IRR (95% CI)			
		Female	Male	10–25	26–40	40–55	56+
Non-migrant hh	1	1	1	1	1		1
Female migrant hh	1.60 (1.38, 1.85)	1.52 (1.24, 1.86)	1.74 (1.41, 2.14)	1.55 (1.28, 1.87)	1.69 (1.26, 2.27)	2.17 (1.39, 3.39)	1.98 (0.90, 4.36)
Male migrant hh	1.01 (0.76, 1.35)	1.06 (0.71, 1.60)	0.94 (0.63, 1.41)	1.00 (0.68, 1.47)	1.04 (0.64, 1.71)	0.89 (0.32, 2.46)	0.54 (0.07, 3.93)
Male & Female migrant hh	0.62 (0.29, 1.34)	0.47 (0.15, 1.49)	0.79 (0.29, 2.14)	0.55 (0.2, 1.53)	0.31 (0.04, 2.25)	1.16 (0.15, 8.94)	2.93 (0.4, 21.43)

^a^Adjusted for age, sex, and intervention arm; ^b^Adjusted for age and intervention arm [p-value for interaction with age = 0.69]; ^c^Adjusted for sex and intervention arm [*p*-value for interaction with sex = 0.73]

### Results from the secondary analysis

Adjusting for the educational attainment of the head of the household (marker of household level SEP - Model B) and household level ‘problem’ alcohol use (Model C) slightly attenuated the associations observed (Table 3). Both poorer household level socioeconomic position and ‘problem’ alcohol use were independently associated with an increased risk of attempted suicide. We explored the association with suicide as a secondary outcome (*n* = 120) and found no statistical evidence of an elevated risk in migrant households (IRR (95% CI) – female migrant 0.95 (0.53, 1.69); male migrant 1.08 (0.34, 3.41); male & female migrant 2.04 (0.28, 14.7)), though the study is underpowered to detect anything but large differences between the migration status of households and suicide.

**Table 3:** Risk of attempted suicide by household (hh) migrant status adjusted for potential confounder/mediating variables

	Models - IRR (95% CI)				
	A	B	C	D	E
Migration status					
Non-migrant hh	1	1	1	1	1
Female migrant hh	1.69 (1.45, 1.97)	1.48 (1.28, 1.72)	1.59 (1.37, 1.84)	1.69 (1.45, 1.95)	1.57 (1.35, 1.82)
Male migrant hh	1.03 (0.76, 1.39)	1.00 (0.75, 1.34)	1.02 (0.77, 1.37)	1.07 (0.80, 1.44)	1.09 (0.82, 1.46)
Male & Female migrant hh	0.60 (0.26, 1.36)	0.58 (0.27, 1.24)	0.62 (0.29, 1.32)	0.73 (0.34, 1.57)	0.69 (0.32, 1.48)

Model A – Age, sex and intervention arm adjusted

Model B - Age, sex, intervention arm and education of head of household adjusted

Model C - Age, sex, intervention arm and ‘problem’ household alcohol status adjusted

Model D - Age, sex, intervention arm and household size adjusted

Model E –Age, sex, intervention arm, education of head of household, ‘problem’ household alcohol status and household size adjusted

### Results from the sensitivity analysis

As a sensitivity analysis we investigated whether the inclusion of graduate migrants into the migrant household category altered the associations observed. The associations were slightly attenuated but were consistent with an elevated risk of attempted suicide in migrant households.

## Discussion

### Main findings

Living in a household with a foreign migrant household member (measured at the start of the follow-up period) was associated with an increased risk of attempted suicide, with evidence that the risk was elevated in households with a female working abroad, but not in households with a male working abroad.

### Strengths and limitations

This is the first large scale longitudinal investigation of the association between household experience of temporary foreign migration and a mental health outcome in a LMIC. The study benefits from a comprehensive surveillance system of attempted suicide and a high baseline response rate (95%). The results of this study, however, should be interpreted in light of dataset limitations. First, the baseline survey had limited emigration details. For example, we only recorded whether there was a current migrant in the household, regardless of the duration or destination. We also do not have data on the type of migration (i.e. skilled or unskilled), though we tried to identify this based on the education level of the migrant worker. We also do not know exactly who migrated (e.g. mother/father). We attempted to overcome this limitation by classifying the migrant according to their sex (as inter-relationships between household members were not recorded) and investigating whether there was a differential effect of the migration on the age/sex of the left-behind family member. We were also unable to investigate the potential mitigating factors, such as frequency of communication, remittances, (i.e. money sent home by the migrant worker) and household composition, on the association between being a left-behind family member and suicidal behaviour. This is especially important as previous retrospective studies have found that these factors are important in reducing the impact of the migration on the mental health outcomes of left-behind family members [13, 24]. Second, we were only able to investigate the association between current migrant households with suicidal behaviour for those left-behind in the household where the migrant worker was a resident. Given that children are sometimes left in the care of other relatives (other than the spouse, which is especially the case when the mother migrates), the resident status of the child may be misclassified as being in a non-migrant household. Third, we recorded the migration status of the household at the start of the study period and assumed that the migration status of the household did not change during the follow-up period. This is an important limitation as it may be that the suicide attempt occurred when the migrant returned home as opposed to whilst they were away, as has been previously reported. Disputes can arise when the migrant returns home and finds that remitted money has been mismanaged or discover that the left-behind spouse has engaged in an extramarital affair. These discoveries have been linked to suicide in previous ethnographic work [4]. Therefore, it is possible that the link we observe in this study is not a consequence of the left-behind environment, but due to return migration. Fifth, we have focused on a single health outcome (suicidal behaviour), but the balance of benefits and risks of being a left-behind family member is complex. Investigations of the impact of temporary migration needs to take into account a range of health outcomes, as well as short- and long-term harms and benefits. Lastly, we do not have data on the drivers for migration, nor on factors which were present prior to the migration. For example, in this context women may choose to emigrate to escape abusive domestic situations or poverty [25]. Therefore, the observed associations may be a consequence of factors that were present before, and may have precipitated, the migration and may persist post-migration (e.g. household violence, poverty). Given the limited number of potential confounding measures we were able to control for, it is possible (especially given the magnitude of the associations) that the observed relationship has arisen purely due to confounding.

### Comparison to other studies

Consistent with previous research from Sri Lanka [11, 26, 27], we find that left-behind family members have a higher risk of poorer mental health outcomes, though none of these studies investigated suicidal behaviour. A small Jamaican case-control study investigated the link between parental emigration and suicidal ideation in left-behind offspring but found no evidence of an increased risk (OR 1.4 95% CI 0.2, 10.5) [28]. The study was underpowered to detect any difference in suicidal ideation as it only included 54 young children (9–10-year olds).

Much of the research in left-behind families has focused on investigating the impact of parental migration on the health of left-behind children, with a large proportion of these studies investigating internal migration (particularly within China). Internal migration is the movement of people within a country, whilst international migration is movement across international borders. It has been argued that the experience of being left-behind is different for households with internal vs. international migrants, with worse outcomes in left-behind families of international migrants [29, 30]. Evidence from internal migration studies indicate that both left-behind children [31, 32] and adult family members [33] experience poorer mental health, though not all studies find an elevated risk [34, 35]. This is consistent with international migration studies which find statistical evidence of poorer mental health in family members left-behind [11, 26, 36], as well as null associations [37, 38].

Maternal emigration [39] and migration to the Middle East [40] is associated with worse outcomes in left-behind family members. This is further supported by the current study which shows a higher rate of attempted suicide in households with a female migrant (92% of migrating Sri Lankan women emigrate to the Middle East [18]), but no evidence of an elevated risk in male migrant households. A possible explanation for the elevated risk in female migrant households might be that women are usually the primary caregivers in a household and therefore their absence is more disruptive to day-to-day caregiving arrangements. The reorganisation of duties needed for women to migrate may require male members of the household to adopt new gender roles; a change from the breadwinner to taking over “women’s work” [4]. In a strongly patriarchal society, like Sri Lanka, this can result in decreased attention to children’s needs and a loss of self-respect. Men sometimes attempt to reinstate this loss of status by buying alcohol for male relatives and friends, and to manage their anxieties with the heavy consumption of alcohol [41]. Whilst controlling for ‘problem’ alcohol use in the household reduced the strength of the association (by 10%) between being from a household with a female migrant and an increased risk of attempted suicide, the migratory status of the household was still related to a 59% increased risk, suggesting that other influences may also be important. Further work is needed to understand the mechanisms underlying this complex association to help identify targets for intervention.

## Conclusions

We find that individuals in households with a female foreign migrant are at an increased risk of attempted suicide, but findings must be interpreted with caution. The increased risk of suicidal behaviour in these households may be due to factors that were present before the migration and may have prompted it (e.g. household violence, poverty). There has been significant concern over the well-being of children left behind by migration, and the current study provides further evidence to support this concern. However, we also identified that the observed vulnerability is not limited to children, but to all left-behind family members. Despite the strength of the prospective design of this study, the relationship between migration and mental health is still poorly understood. Further longitudinal studies are needed to extend our understanding and identify potential mitigating factors which could help reduce the risk of attempted suicide in left-behind family members.

## Additional file

Additional file 1: Characteristics of participants with missing data and crude risk of attempted suicide by household migrant status. **Table S1.** Number of participants with missing data for each variable of interest. **Table S2.** Unadjusted risk of attempted suicide by household (hh) migrant status. (DOCX 15 kb)

## Abbreviations

CI: Confidence interval
IRR: Incidence rate ratio
LMIC: Low and middle income country
SD: Standard deviation
