# Supplementary material for: Are left-behind families of migrant workers at increased risk of attempted suicide? – a cohort study of 178,000+ individuals in Sri Lanka
Source: BMC Psychiatry. 2019 Jan 15;19:25. doi: 10.1186/s12888-018-2000-8 (PMC6332866; doi:10.1186/s12888-018-2000-8)
Supplement: Supplementary file 1 — Characteristics of participants with missing data and crude risk of attempted suicide by household migrant status. Table S1. Number of participants with missing data for each variable of interest. Table S2. Unadjusted risk of attempted suicide by household (hh) migrant status. (DOCX 15 kb) [file 12888_2018_2000_MOESM1_ESM.docx]

Table S1: Number of participants with missing data for each variable of interest

|  |  |  |
| --- | --- | --- |
|  |  | Missing data n (%) |
| N= | | 821 (0.46) |
|  | |  |
| Household measures | |  |
| Problem' alcohol use | | 56 (0.03) |
|  |  |  |
| Individual measures | |  |
| Sex | | 56 (0.03) |
| Age (years) | | 304 (0.17) |
| Education of head of household | | 430 (0.24) |
| Frequency at home | | 103 (0.06) |

Table S2 Unadjusted risk of attempted suicide by household (hh) migrant status

|  | Crude IRR (95% CI) |
| --- | --- |
| Non-migrant hh | 1 |
| Female migrant hh | 1.68 (1.44, 1.95) |
| Male migrant hh | 0.95 (0.71, 1.27) |
| Male & Female migrant hh | 0.61 (0.28, 1.33) |
